# Specific growth rate and substrate dependent polyhydroxybutyrate production in *Saccharomyces cerevisiae*

**DOI:** 10.1186/2191-0855-3-18

**Published:** 2013-03-21

**Authors:** Kanokarn Kocharin, Jens Nielsen

**Affiliations:** 1 Department of Chemical and Biological Engineering, Chalmers University of Technology, Kemivägen 10, Göteborg, SE-41296, Sweden

**Keywords:** Polyhydroxybutyrate, *Saccharomyces cerevisiae*, Specific growth rate

## Abstract

Production of the biopolymer polyhydroxybutyrate (PHB) in *Saccharomyces cerevisiae* starts at the end of exponential phase particularly when the specific growth rate is decreased due to the depletion of glucose in the medium. The specific growth rate and the type of carbon source (fermentable/non-fermentable) have been known to influence the cell physiology and hence affect the fermentability of *S. cerevisiae*. The production of PHB utilizes cytosolic acetyl-CoA as a precursor and the *S. cerevisiae* employed in this study is therefore a strain with the enhanced cytosolic acetyl-CoA supply. Growth and PHB production at different specific growth rates were evaluated on glucose, ethanol and a mixture of glucose and ethanol as carbon source. Ethanol as carbon source yielded a higher PHB production compared to glucose since it can be directly used for cytosolic acetyl-CoA production and hence serves as a precursor for PHB production. However, this carbon source results in lower biomass yield and hence it was found that to ensure both biomass formation and PHB production a mixture of glucose and ethanol was optimal, and this resulted in the highest volumetric productivity of PHB, 8.23 mg/L · h^-1^, at a dilution rate of 0.1 h^-1^.

## Introduction

*Saccharomyces cerevisiae* is a biotechnologically important microorganism. The well-established knowledge and the availability of genome data have led to its versatile use as a cell factory for many industrial products (Ostergaard et al. 2000). Process optimization for production of various industrial products such as biofuels, fine and bulk chemicals in *S. cerevisiae* has been studied by several research groups (de Jong et al. 2012; Hong and Nielsen 2012; Nevoigt 2008; Ostergaard et al. 2000; Steen et al. 2008). This reveals the physiological adaptability of *S. cerevisiae* to a highly variable environment. According to a respiratory-fermentative metabolism in *S. cerevisiae*, the type (fermentable/non fermentable) and concentration of carbon source as well as the availability of oxygen are important factors driving the metabolic pattern in the yeast. In order to improve productivity for any products in *S. cerevisiae*, it is important to know the relationship between growth and product formation.

The bacterial PHB biosynthesis pathway has previously been introduced into the yeast’s genome and *S. cerevisiae* has been evaluated as a cell factory for PHB production (Breuer et al. 2002; Dimster-Denk and Rine 1996; Leaf et al. 1996; Marchesini et al. 2003; Zhang et al. 2006). The production of PHB in *S. cerevisiae* starts at the end of the exponential growth phase specifically when glucose is depleted from the medium (Carlson et al. 2002; Kocharin et al. 2012). From our earlier work, we demonstrated that PHB production can be improved by co-transformation of the plasmid containing the PHB biosynthesis pathway with an acetyl-coenzyme A (acetyl-CoA) boost plasmid designated to improve the availability of cytoplasmic acetyl-CoA (2012). However, a difference was observed in the productivity when the production was scaled up from shake flasks to bioreactor cultivations. We suspected that the higher specific growth rate obtained in the bioreactor has an effect on PHB production. It is known that the specific growth rate influences the physiology of *S. cerevisiae* hence affecting the fermentative capacity, respiratory metabolism and other metabolic activities (Blank and Sauer 2004; Frick and Wittmann 2005; Van Hoek et al. 1998). Therefore, the difference in specific growth rate is hypothesized to be responsible for the lower PHB production in the bioreactor cultivation. In the present study, we employ a chemostat cultivation system to investigate PHB production at different dilution rates (which correspond to different specific growth rates). Furthermore, we assess PHB production in *S. cerevisiae* grown on different carbon sources, glucose, ethanol and a mixture of glucose and ethanol.

## Materials and methods

### Strains and pre-culture conditions

*S. cerevisiae* harboring the acetyl-CoA boost plasmid and the PHB plasmid (SCKK006) was used in this study. The acetyl-CoA plasmid contained four genes; alcohol dehydrogenase (*ADH2*) and acetaldehyde dehydrogenase (*ALD6*), acetyl-CoA C-acetyltransferase (*ERG10*) and acetyl-CoA synthetase (acs^L641P^) from *Streptococcus mutans*. The details in the acetyl-CoA boost plasmid are described by Chen and co-workers (Chen et al. 2013). The PHB plasmid (pKK01) contained three PHB genes from *Ralstonia eutropha*, *PhaA* (β-ketothiolase), *PhaB* (acetoacetyl-CoA reductase) and *PhaC* (polyhydroxyalkanoate synthase). All of the heterologous genes were codon optimized for better expression in *S. cerevisiae*. The details on strain construction have been described previously (Kocharin et al. 2012).

The pre-cultures for bioreactor cultivations were prepared by inoculation of 5 mL of a defined minimal medium in a 14 mL culture tube with a single colony and grown at 30°C and 180 rpm in an orbital shaking incubator. After 15 h, the culture was transferred into 50 mL of defined minimal medium in a 500 mL baffled flask and grown at 30°C with 180 rpm in an orbital shaking incubator. The minimal medium for pre-culture cultivations had the same composition as the medium used for bioreactor cultivation.

### Chemostat bioreactor cultivation

PHB production was evaluated in defined minimal media (Verduyn et al. 1992) prepared as follows (per liter): (NH_4_)_2_SO_4,_ 5 g; KH_2_PO_4_, 3 g; MgSO_4_⋅7H_2_O, 0.5 g; trace metal solution, 1 mL; and vitamin solution,1 mL, with an initial pH of 6.5. Glucose was autoclaved separately from the minimal medium and later added to the media at the concentration of 20 g/L. The trace metal solution consisted of the following (per liter): EDTA (sodium salt) 15 g; ZnSO_4_⋅7H_2_O, 0.45 g; MnCl_2_⋅2H_2_O, 1 g; CoCl_2_⋅6H_2_O, 0.3 g; CuSO_4_⋅5H_2_O, 0.3 g; Na_2_MoO_4_⋅2H_2_O, 0.4 g; CaCl_2_⋅2H_2_O, 0.45 g; FeSO_4_⋅7H_2_O, 0.3 g; H_3_BO_3_, 0.1 g and KI, 0.1 g. The pH of the trace metal solution was adjusted to 4.0 with 2 M NaOH. The vitamin solution contained (per liter): biotin, 0.05 g; ρ-amino benzoic acid, 0.2 g; nicotinic acid, 1 g; Ca-pantothenate, 1 g; pyridoxine-HCl, 1 g; thiamine-HCl, 1 g and myo-inositol, 25 g. The pH of the vitamin solution was adjusted to pH 6.5 prior filter sterilization.

The bioreactor was inoculated with an amount of pre-culture that resulted in a final OD_600_ of 0.02. When the glucose and ethanol during batch cultivation was almost completely consumed, the feeding systems for the chemostat operations were started. The aerobic chemostat was performed in 1.0 L stirrer-pro vessels (DasGip, Jülich, Germany) with a working volume of 0.5 L. The temperature was controlled at 30°C using a bioBlock integrated heating and cooling thermo well. Agitation was maintained at 600 rpm using an overhead drive stirrer with one Rushton impeller. The air flow rate was kept at 1 vvm. The pH was maintained constant at 5.0 by the automatic addition of 2 M KOH. Dissolved oxygen was monitored and maintained above 30% saturation. All the feed media had the same composition and were prepared as described above except for the carbon source. The carbon sources used were 100% glucose, 100% ethanol and a mixture of glucose and ethanol at the ratio of 1:2. The carbon sources in the feed medium were prepared based on the C-molar concentration of 20 g/L glucose (0.666 Cmol/L) as in the medium used during batch cultivation. Therefore, feed media with 15.32 g/L ethanol, and a mixture of 6.35 g/L glucose and 10.21 g/L of ethanol were prepared yielding a final carbon concentration of 0.666 Cmol/L. To obtain a dilution rate of 0.05 h^-1^, 0.1 h^-1^, 0.15 h^-1^ and 0.2 h^-1^, the inlet medium was fed at 25 ml/h, 50 mL/h, 75 mL/h and 100 mL/h respectively. Samples were taken when the fermentation reached the steady state, defined by constant values of carbon dioxide transfer rate (CTR), oxygen transfer rate (OTR) and biomass concentration.

### Cell mass determination

Culture samples of 10 mL volume were centrifuged at 5,000 rpm and 4°C for 5 min and the pellets were washed once with distilled water and centrifuged at 14,000 g for 1 min. The recovered cell pellet was immediately frozen by immersion in liquid nitrogen followed by lyophilization under vacuum (Christ Alpha 2–4 LSC, Shropshire, UK). The dry cell weight was determined and the pellet kept at 4°C for further analysis.

### Metabolite analysis

Metabolites including glucose, ethanol, glycerol, and acetate were quantified in the culture supernatant using an Ultimate 3000 HPLC (Dionex, Sunnyvale, CA, USA) equipped with an Aminex HPX 87H ion exclusion column (300 mm × 7.8 mm, Bio-Rad Laboratories, Hercules, CA, USA) which was operated at 45°C and a flow rate of 0.6 mL/min of 5 mM H_2_SO_4_ using a refractive index detector and UV detector for analysis of sugars and organic acids, respectively.

PHB was analyzed as described previously (Karr et al. 1983; Tyo et al. 2006). 10–20 mg of dried cells were weighed and boiled in 1 mL of concentrated sulfuric acid for 60 min and then diluted with 4 mL of 14 mM H_2_SO_4_. Samples were centrifuged (15 min, 16,000 × g) to remove cell debris, and the supernatant was analyzed using an Ultimate 3000 HPLC (Dionex) equipped with an Aminex HPX-87H ion exclusion column (300 × 7.8 mm; Bio-Rad Laboratories) and UV detector. Commercially available PHB (Sigma-Aldrich, St. Louis, MO), processed in parallel with the samples, was used as a standard. The HPLC was operated at 60°C and a flow rate of 0.6 mL/min of 5 mM H_2_SO_4_.

## Results

### PHB production at different dilution rates

In this study, we employed a chemostat cultivation technique to investigate PHB production at different specific growth rates of an engineered *S. cerevisiae* strain, SCKK006, in which genes from the ethanol degradation pathway were overexpressed in order to enhance the supply for acetyl-CoA used as a precursor for PHB production. Besides, we also investigated the production of PHB when the engineered strain was grown on different carbon sources. In batch cultures, the maximum specific growth rate of SCKK006 on glucose in a defined minimal medium with 20 g/L glucose as carbon source was 0.34 h^-1^. Kinetic parameters and yields during batch cultivation are reported in Table 1. The ethanol yield and glycerol yield on glucose were 0.2293 Cmol/Cmol and 0.0241 Cmol/Cmol, respectively. Detectable amounts of pyruvate, succinate and acetate were observed during the batch cultivation. When the ethanol was almost completely depleted from the medium (observed by a drop of the carbon dioxide profile measured from the exhaust gas via a gas analyzer), the chemostat cultivation was started.

**Table 1:** Yields and kinetic parameters obtained from batch cultivations

	**μ**	**r**_**s**_	**Y**_**sx**_	**Y**_**sEtOH**_	**Y**_**sGly**_	**Y**_**sPyr**_	**Y**_**sAce**_	**Y**_**sSuc**_
	**h**^-1^	**mmol/gDW · h**^-1^	**g/Cmol**	**Cmol/Cmol**	**Cmol/Cmol**	**Cmol/Cmol**	**Cmol/Cmol**	**Cmol/Cmol**
Mean	0.34	3.79	1.84	0.2293	0.0241	0.0033	0.0064	0.0033
SD	0.01	0.52	0.26	0.01	0.00	0	0	0

The values were calculated from at least triplicate fermentations (n ≥ 3) and are represented as mean ± SD.

μ = specific growth rate; r_s_ = specific substrate uptake rate; Y_sx_ = biomass yield on substrate; Y_sEtOH_ = ethanol yield on substrate; Y_sGly_ = glycerol yield on substrate; Y_sPyr_ = pyruvate yield on substrate; Y_sAce_ = acetate yield on substrate; _YsSuc_ = succinate yield on substrate.

The chemostat cultivation was operated at different dilution rates ranging from 0.05 h^-1^ to 0.2 h^-1^ with the feed medium containing different carbon sources. The physiological parameters are reported in Table 2. When the feed medium contained glucose as the sole carbon source, ethanol was detected when the dilution rate was higher than 0.1 h^-1^. This reveals a respiro-fermentative metabolism of *S. cerevisiae* grown at high dilution rate or high sugar content even in aerobic cultivation with excess oxygen (Hanegraaf et al. 2000). At a dilution rate higher than 0.15 h^-1^, a very small amount of glycerol (<10 mg/L) was observed. The highest biomass yield, 0.57 Cmol/Cmol was found at a dilution rate of 0.1 h^-1^. At dilution rates higher than 0.1 h^-1^, the biomass yield tended to decrease. The highest PHB yield was also observed at a dilution rate of 0.1 h^-1^, 3.67 Cmmol/Cmol substrate.

**Table 2:** Yields and kinetic parameters obtained during chemostat cultivations

**Feeding component**	**Dilution rate**	**Y**_**sx**_	**Y**_**sEtOH**_	**Y **_**sPHB**_	**Ethanol accumulation**	**Y**_**xPHB**_
	**h**^**-1**^	**Cmol/Cmol**	**Cmol/Cmol**	**Cmmol/Cmol**	**Cmol/L**	**mg/gDW**
Glucose	0.05	0.51 ± 0.01	-	2.51 ± 0.07	0	4.33 ± 0.19
	0.10	0.57 ± 0	-	3.67 ± 0	0	5.59 ± 0
	0.15	0.36 ± 0.02	0.02 ± 0	2.44 ± 0	-	5.94 ± 0.64
	0.20	0.29 ± 0	0.1 ± 0	1.92 ± 0	-	5.59 ± 0.23
Ethanol	0.05	0.45 ± 0.01	0	8.50 ± 0.23	0	16.55 ± 0.02
	0.10	0.37 ± 0	-	4.94 ± 0.58	0.0964 ± 0	13.49 ± 0
	0.15	0.12 ± 0.01	-	0.46 ± 0.05	0.2364 ± 0	5.30 ± 0.31
Glucose: Ethanol (1:2)	0.05	0.48 ± 0.02	0	9.97 ± 0.07	0	18.34 ± 0.53
	0.10	0.38 ± 0	-	7.41 ± 0	0.2012	12.41 ± 0
	0.15	0.30 ± 0	-	2.57 ± 0.1	0.1637	7.50 ± 0.19
	0.20	0.22 ± 0	-	1.13 ± 0	0.2769	4.56 ± 0

The values were calculated from duplicate fermentations and are represented as mean ± SD. The formula for biomass used in this calculation is CH_1.8_O_0.5_ N_0.2_. Y_sx_ = biomass yield on substrate, Y_sEtOH_ = ethanol yield on substrate, Y_sPHB_ = PHB yield on substrate, Y_xPHB_ = PHB yield per biomass.

When the feed medium contained ethanol as the sole carbon source, the highest biomass yield and PHB yield on substrate, 0.45 Cmol/Cmol and 8.5 Cmmol/Cmol, was observed when the chemostat was operated at the dilution rate of 0.05 h^-1^. The biomass yield and the PHB yield tended to decrease when increasing the dilution rate. At dilution rates higher than 0.05 h^-1^, ethanol accumulated and progressively increased in the medium when the dilution rate was increased. When the chemostat was operated at 0.15 h^-1^, after 5 residence times, the amount of ethanol accumulated in the medium almost reached the level of ethanol in the feed. When the chemostat was operated at a dilution rate of 0.2 h^-1^, the biomass decreased and became zero due to washout over 3 resident times.

When glucose or ethanol were used as carbon source, the biomass yield and PHB yield on the mixed-substrate in the feed medium was calculated based on the C-moles of consumed substrate. The maximum biomass yield, 0.48 ± 0.02 Cmol/Cmol, and the maximum PHB yield, 9.97 ± 0.07 Cmmol/Cmol, were obtained when the mixed-substrate was fed at 0.05 h^-1^. Moreover, no accumulation of ethanol was observed when the chemostat was operated at 0.05 h^-1^ while 0.16-0.28 Cmol/L of ethanol were observed when the chemostat was operated at dilution rates higher than 0.05 h^-1^. The amount of accumulated ethanol on the mixed-substrate was similar to the amount of ethanol accumulated in the medium when the chemostat was fed with ethanol alone before the washout occurred The biomass yield on substrate was substantially decreased when the dilution rate was increased on all substrates used in this study.

### PHB production in *S. cerevisiae* grown on different carbon sources

**Figure 1:**
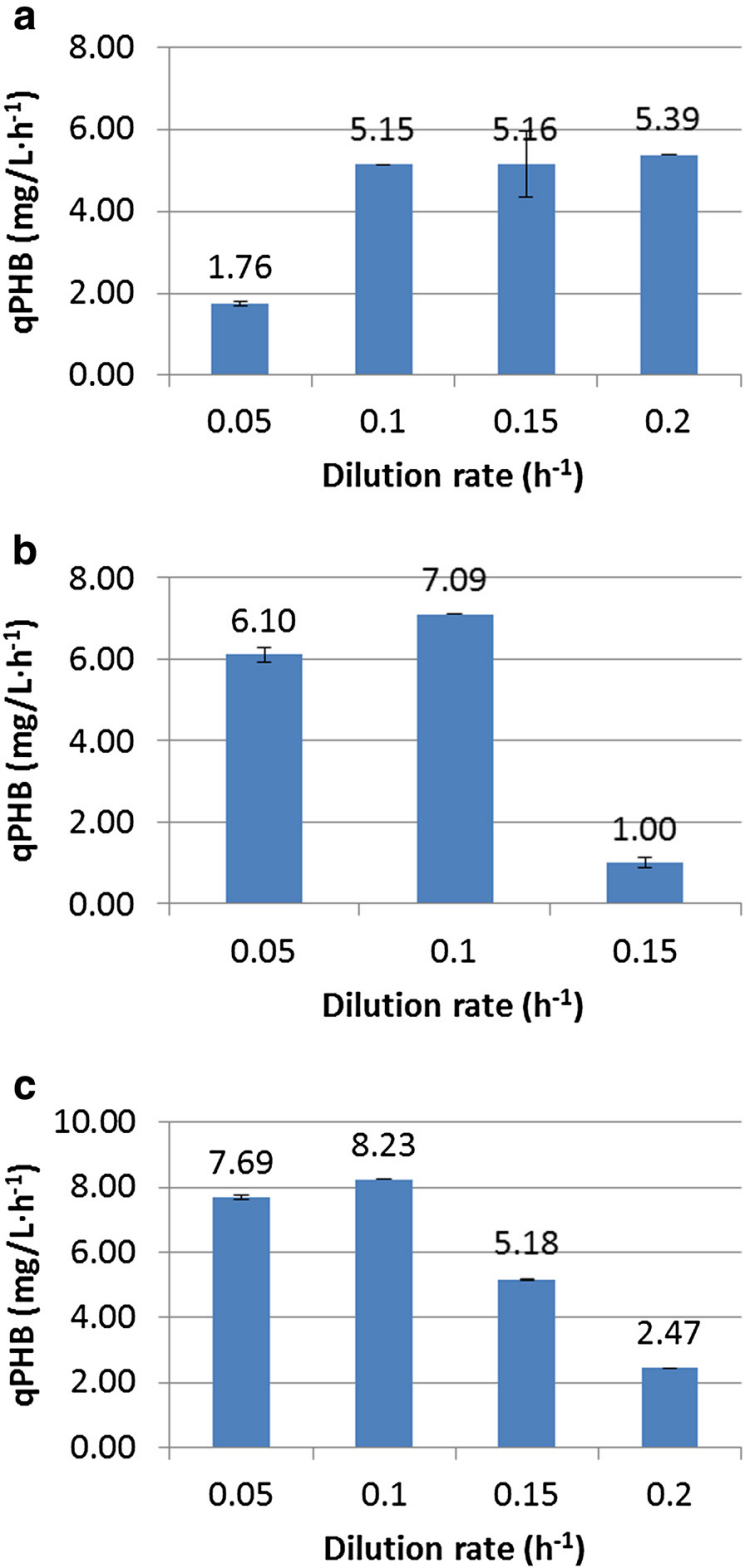
**The PHB productivity productivities of recombinant PHB in *S. cerevisiae *grown on different substrates at different dilution rates from the chemostat cultivation. a**) Glucose, **b**) Ethanol, **c**) Mixed-substrate.

Comparing substrate utilization at the same dilution rate, glucose showed the highest biomass yield on substrate at all dilution rates investigated in this study, followed by the mixed-substrate and ethanol, respectively. The maximum PHB content, 18.34 mg/gDW, was obtained when the mixed substrate was used in the feed at a dilution rate of 0.05 h^-1^. Comparing either glucose or ethanol as sole carbon source, ethanol alone resulted in a ~3 times higher PHB yield when the chemostat was operated at D = 0.05 h^-1^ although it led to a lower biomass yield on substrate. When the feed medium contained glucose and ethanol as carbon sources, the highest PHB yield was obtained compared to using a single carbon source as a substrate. Comparing the overall volumetric productivities in Figure 1, the mixed-substrate at a dilution rate of 0.1 h^-1^ revealed the highest volumetric productivity of 8.23 mg/L · h^-1^. When ethanol was produced during growth on glucose or was accumulated when using ethanol or the mixed-substrate containing ethanol as a feed medium, the volumetric productivity did not improve. However, when glucose was fed at a dilution rate of 0.2 h^-1^, a slight increased in the volumetric productivity of PHB was observed.

## Discussion

**Figure 2:**
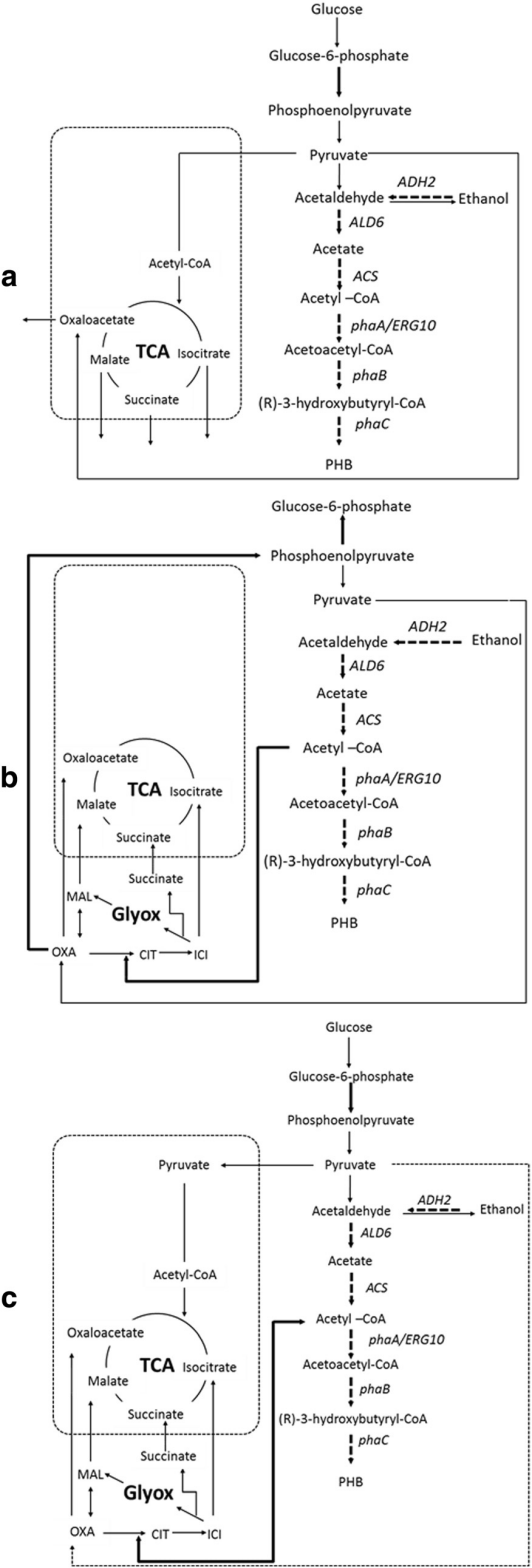
**Central carbon metabolism of PHB producing *S. cerevisiae *grown on different substrates. a**) Glucose, **b**) Ethanol and **c**) Mixed-substrate of glucose and ethanol. *ADH2* = alcohol dehydrogenase; *ALD6* = aldehyde dehydrogenase; *ACS* = acetyl-CoA synthetase (acs^L641P^); *ERG10* = acetyl-CoA C-acetyltransferase; *PhaA* (β-ketothiolase); *PhaB* (acetoacetyl-CoA reductase); *PhaC* (polyhydroxyalkanoate synthase). *ICI* = isocitrate; *CIT* = citrate: *OXA* = oxaloacetate; *MAL* = malate; *Glyox* = glyoxylate.

In *S. cerevisiae*, high specific growth rate and high sugar concentration trigger the production of ethanol, even during fully aerobic cultivation. In this study, when the chemostat was operated at dilution rates of 0.15 h^-1^ and 0.2 h^-1^, ethanol was produced in the medium as an evidence of a respiro-fermentative metabolism of *S. cerevisiae*, which can ferment glucose at a high dilution rate (Duntze et al. 1969; Hanegraaf et al. 2000; Maaheimo et al. 2001). When using glucose as a carbon source in the feed medium, a high specific growth rate (corresponding to a high dilution rate) reduces the flux distribution to the pentose phosphate pathway, which might lower the NADPH concentration (Frick and Wittmann 2005). Since NADPH is required in the PHB biosynthesis pathway, higher specific growth rates might influence the availability of NADPH and thus substantially lower PHB production. Moreover, production of PHB consumes cytosolic acetyl-CoA which is in this case produced mainly from the overexpression of the ethanol degradation pathway (Figure 2a). Therefore, the PHB yield on glucose was lower than the PHB yield on ethanol. Besides, a small amount of glycerol was produced in order to maintain the redox balance when ethanol was produced from glucose feeding at high dilution rates. In this study, a lower biomass yield on ethanol was observed compared to the biomass yield on glucose at all dilution rates. However, the PHB yield on ethanol was higher compared with using only glucose in the feed medium. This might be due to the fact that ethanol in the feed replaces pyruvate as the direct source for cytosolic acetyl-CoA production and hence results in a higher Y_sPHB_ compared to with glucose as carbon source (Figure 2b). When *S. cerevisiae* is grown on ethanol, the synthesis of biomass and the TCA intermediates requires activity of the glyoxylate and gluconeogenesis pathways (de Jong-Gubbels et al. 1995; Maaheimo et al. 2001) and this explains the lower biomass yield compared to glucose. When *S. cerevisiae* is grown on the glucose and ethanol mixture, glucose is used as carbon and energy source for growth whereas ethanol can serve as the main source for cytosolic acetyl-CoA production and hence the carbon source for PHB production (de Jong-Gubbels et al. 1995) (Figure 2c). Therefore, the mixed-substrate used in this study resulted in higher biomass and PHB yields on substrate compared to the use of a single substrate in the feed medium. However, when the mixed-substrate was fed at a dilution rate higher than 0.05 h^-1^, ethanol started to accumulate ranging from 0.16 Cmol/L to 0.2 Cmol/L. The accumulated ethanol at higher dilution rates affected the ratio of glucose and ethanol in the bioreactor, thus influencing the overall regulation of the central carbon metabolism (de Jong-Gubbels et al. 1995) and eventually resulting in washout of the cells from the continuous bioreactor. In conclusion, ethanol is a better carbon source for PHB production in yeast compared to glucose since it can be directly used for acetyl-CoA production and hence serve as a precursor for PHB production. In terms of productivity, to compromise between growth of *S. cerevisiae* and PHB production, feeding of a mixed-substrate at the appropriate ratio between glucose and ethanol, where no ethanol accumulate in the medium, i.e. the chemostat operated at a dilution rate of 0.1 h^-1^ results in maximum PHB production.

## Competing interests

The authors declare that they have no competing interest.

## Authors’ contributions

KK and JN participated in the design of the experiment. KK performed all the experiments, analyzed the data and wrote the manuscript. JN edited the manuscript. All authors read and approved the final manuscript.
